# Associations between peripheral nerve stimulation and cognitive performance: insights from healthy individuals and various disease pathologies

**DOI:** 10.3389/fnagi.2025.1518198

**Published:** 2025-09-02

**Authors:** Xing Liu, Chenyi Yang, Xinyi Wang, Zixuan Wang, Huihui Liao, Huan Liu, Miao Zhang, Lin Zhang, Haiyun Wang

**Affiliations:** ^1^The Third Central Clinical College of Tianjin Medical University, Tianjin, China; ^2^Department of Anesthesiology, Central Hospital, Tianjin University, Tianjin, China; ^3^Tianjin Key Laboratory of Extracorporeal Life Support for Critical Diseases, Tianjin, China; ^4^Nankai University, Tianjin, China; ^5^Tianjin University, Tianjin, China; ^6^Nankai University Affinity the Third Central Hospital, Tianjin, China

**Keywords:** peripheral nerve stimulation, cognitive function, healthy individuals, Alzheimer’s disease, epilepsy

## Abstract

Cognitive dysfunction can manifest as declines in memory, learning, and attention, stemming from multifaceted factors. Risk factors encompass a spectrum including genetics, lifestyle choices, and personal medical history. Conditions such as Alzheimer’s disease (AD), depression, epilepsy, and exposure to surgical/anesthesia may correlate with cognitive impairment. Recent advancements in nerve stimulation techniques indicate significant potential for enhancing cognitive function. Understanding the mechanisms of peripheral nerve stimulation (PNS) can improve the management of cognitive impairment and promote its clinical application, advancing cognitive rehabilitation for patients. Following a comprehensive search and selection process, we finally included 47 studies that examined the effects of PNS on cognitive performances of both healthy individuals and various disease pathologies. The aggregated findings suggest that PNS influences crucial brain pathways, such as the ganglia and nucleus tractus solitarius, which project to areas essential for memory consolidation, including the hippocampus and amygdala. PNS improves cognitive function through mechanisms such as neurotransmitter modulation and neuronal activity regulation. However, the effects of PNS on cognitive function vary depending on the pathological condition. Additionally, the efficacy of PNS is influenced by both the intensity and pattern of stimulation. In summary, PNS appears to be a promising modality for enhancing cognitive function, particularly in neurological disorders such as AD and epilepsy. While further research is needed to fully elucidate the mechanisms, current evidence suggests that PNS could offer a valuable therapeutic option for improving memory and attention. With its potential for broad application and non-invasive nature, PNS represents an exciting avenue for future research and clinical practice in cognitive enhancement.

## 1 Introduction

Cognitive dysfunction encompasses a range of impairments in memory retention, learning capacity, and attention span, originating from diverse factors including genetic predispositions (gene mutations, familial history of genetic disorders), lifestyle factors (chronic malnutrition, smoking), and personal medical history (aging, head trauma, neuropsychiatric comorbidities, surgical interventions) (Brem and Sensi, 2018; Lai et al., 2022; Nature Review Disease Primer, 2018; Rabin et al., 2017; Wu et al., 2023). The latest findings from the Global Burden of Neurological Disease Survey 2021 underscore the prominence of neurological disorders as the primary contributors to disability-adjusted life years (DALYs), impacting an estimated 443 million individuals worldwide (GBD 2021 Nervous System Disorders Collaborators, 2024). Notable among these disorders are conditions such as stroke, Alzheimer’s disease (AD) and other dementias, and epilepsy. Given the profound impact of neurological disorders on global health, there is an urgent imperative to implement effective preventive, therapeutic, and rehabilitative measures. Despite significant advancements in medical care and the advent of cutting-edge technologies and pharmaceutical interventions, a universally recognized and efficacious approach to ameliorate cognitive dysfunction across all etiologies remains elusive (Nabbout and Kuchenbuch, 2020; Self and Holtzman, 2023). This presents a formidable challenge to patient wellbeing, highlighting the critical need for ongoing research and intervention initiatives.

PNS, encompassing modalities such as vagus and trigeminal nerve stimulation, emerges as a potential solution to this challenge. Among various peripheral nerve stimuli, vagus nerve stimulation (VNS) has garnered substantial attention. Rigorous clinical trials have led to the Food and Drug Administration (FDA) approval for treating refractory epilepsy with PNS (Ben-Menachem et al., 1994; George et al., 1994; Ramsay et al., 1994). Research has documented VNS applications across a spectrum of conditions including epilepsy (Puteikis et al., 2023; Yang et al., 2023), depression (Desbeaumes Jodoin et al., 2018; Sackeim et al., 2001), pain (Busch et al., 2013; Farmer et al., 2020), stroke (Liu et al., 2016; Wu et al., 2018), AD (Merrill et al., 2006; Murphy et al., 2023; Wang et al., 2022), and other cognitive performance related patients (McIntire et al., 2021; Zhang et al., 2020). Initially, due to technological constraints and limited understanding of VNS mechanisms, invasive approaches predominated. This involved implanting neuro cybernetic prosthesis (NCP), a multiprogrammable pulse generator externally programmed with patient-specific stimulation parameters. However, such implantable devices are fraught with drawbacks, including surgical site infections, pulse malfunctions, and associated discomforts such as pain, hoarseness, coughing, and sleep disturbances (Schulze-Bonhage, 2019). Anatomical evidence suggests an extensive vagus nerve distribution within the auricle, hinting at the potential for non-invasive auricular VNS to emulate the effects of implantable VNS. Subsequent transcutaneous auricular vagus nerve stimulation (taVNS) techniques have indeed confirmed this conjecture, underscoring the promise of non-invasive approaches in VNS therapy (Choi et al., 2022; Wang et al., 2022). Similarly, non-invasive stimulation of the trigeminal nerve on the body surface has also shown promise, further expanding the scope of PNS applications (Jonsdottir et al., 2004; Zhang et al., 2020).

**FIGURE 1:**
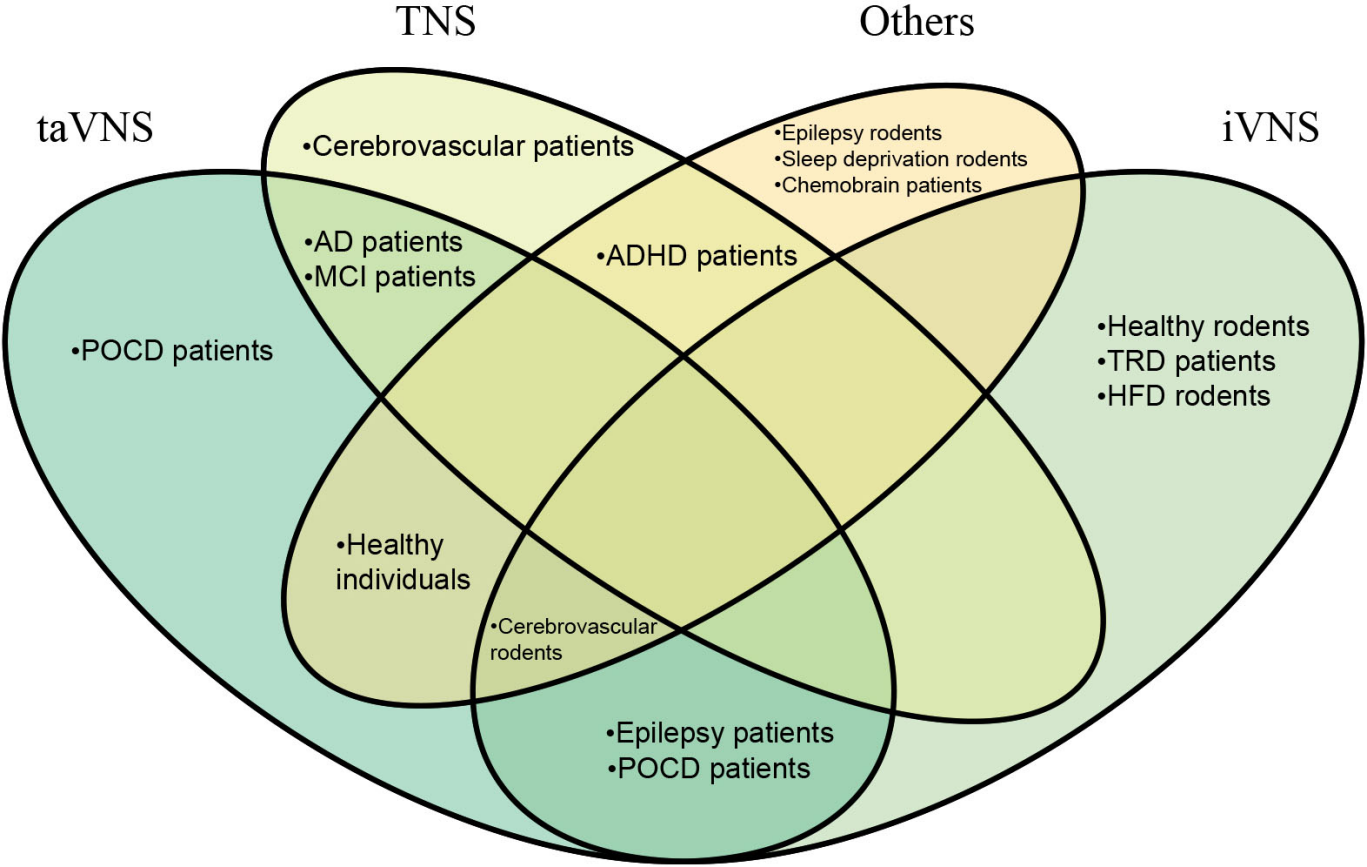
Reported studies on peripheral nerve stimulation in a variety of healthy individuals or pathological conditions.

This paper aims to consolidate existing studies regarding the impact of PNS on cognitive function. This includes elucidating its potential to ameliorate cognitive deficits as well as any potential adverse effects on cognition that may arise during nerve stimulation. Additionally, we will address current challenges in neurostimulation and propose potential solutions. The objective of our study is to provide researchers with a comprehensive understanding of the effectiveness and feasibility of this treatment strategy in preventing or mitigating cognitive impairment, thereby facilitating informed decision-making in future studies. Following a brief classification of healthy individuals and diverse cognitive function-related disorders, we present a distinct review of cognitive performance in response to PNS (Figure 1 and Table 1; see also Supplementary Figure 1 for study selection flowchart).

**TABLE 1:** Characteristics of the included studies.

References	Objective	Disease/model	Peripheral nerve stimulation methods	Output current	Signal frequency	Pulse width	On/off time	Treatment period	Cognitive performance	Risk of bias assessment tools and rating
**1. Healthy individuals**										
De Smet et al., 2023	Human	Healthy individuals	taVNS	–	25 Hz	250	30 s/30 s	20 min	Reduced cognitive rigidity	ROB2 Some concerns
Driskill et al., 2022	Male SD rats	Healthy individuals	iVNS	0.4 mA	30 Hz	500	30 s/150 s	15 min	Improve memory and cognitive flexibility and independent of unspecific changes in locomotion or anxiety.	NA
Fischer et al., 2018	Human	Healthy individuals	taVNS	Average stimulation intensity 1.3 mA	25 Hz	–	–	–	Enhanced ability to adjust to conflicts	ROB2 Some concerns
Olsen et al., 2022	Male SD rats	Healthy individuals	iVNS	0.8 mA	30 Hz	100	18 s/18 s	30 min	Increased NOR and PAT	NA
Sanders et al., 2019	SD rats	Healthy individuals	iVNS	0.8 mA	30 Hz	100 μs	17.5 s/17.5 s	4 days	Improved novelty preference	NA
Sclocco et al., 2020	Human	Healthy individuals	taVNS	Ramp-up output current	2, 10, 25, 100 Hz	300	–	–	100 Hz RAVNS evoked the strongest brainstem response and stronger fMRI responses.	ROBINS-I Moderate
Sommer et al., 2023	Human	Healthy individuals	taVNS	–	25 Hz	250	30 s/30 s	During experiment	Increase in between-task interference in the first test block, but not in subsequent test blocks. No significant on physiological and subjective psychological variables.	ROB2 Some concerns
Tona et al., 2020	Human	Healthy individuals	taVNS	0.5 mA/1 mA	–	–	–	–	No significant cognitive flexibility between groups	ROB2 High risk
Tan et al., 2024	Human	Healthy individuals	Vibrotactile taVNS	–	–	–	–	3 sessions	Improved working memory capacity	ROB2 Some concerns
**2. Alzheimer’s disease and related cognitive impairment**										
Luijpen et al., 2004	Human	MCI patients	TENS	–	160 Hz	100 μs	–	6 weeks	Mild improvement in self-efficacy and mood	ROB2 Some concerns
Luijpen et al., 2005	Human	MCI patients	TENS	–	160 Hz	100 μs	–	6 weeks	No improvement in memory	ROB2 Low risk
Merrill et al., 2006	Human	AD patients	VNS	–	20 Hz	–	30 s/5 min	1 year	12/17 patients did not decline from baseline	ROB2 Some concerns
Murphy et al., 2023	Human	MCI patients	taVNS	10 mA	20 Hz	50 μs	–	–	Improved functional connectivity between semantic and salience function regions	ROB2, Some concerns
Sjögren et al., 2002	Human	AD patients	VNS	0.25 mA	20 Hz	500 μs	30 s/5 min	6 months	7/10 patients responded assessed by ADAS-cog and 9/10 patients responded assessed by MMSE	ROBINS-I Serious
van Dijk et al., 2005	Human	AD patients	TENS	–	160 Hz	100 μs	–	12 weeks	No significant differences on cognitive measures	ROB2 Some concerns
Scherder et al., 1992	Human	AD patients	TENS	Individually adjusted	–	–	–	6 weeks	Improved verbal long-term memory, and verbal fluency improves more in treatment group patients	ROB2 Some concerns
Scherder et al., 1998	Human	AD patients	TENS	–	2/160 Hz	100	–	6 weeks	Improvement in short-term and long term memory, word fluency, and need of help	ROB2 Some concerns
Wang et al., 2022	Human	MCI patients	taVNS	4–6 mA	20/100 Hz	–	–	24 weeks	Increased MoCA-B and AVLT-H, decreased STTB	ROB2 Low risk
**3. Epilepsy**										
Dodrill and Morris, 2001	Human	Epilepsy patients	iVNS	Low and high VNS parameters	–	–	–	12–16 weeks	High stimulation group with higher cognitive flexibility and executive function	ROB2 Some concerns
Ghacibeh et al., 2006	Human	Epilepsy patients	iVNS	0.5 mA	–	–	–	–	Improved cognitive flexibility and creative thinking	ROB2 Some concerns
Hallböök et al., 2005	Human	Refractory epilepsy children	iVNS	0.25 mA	30 Hz	500 μs	30 s/5 min	9 months	No changes in cognitive functioning	ROBINS-I Serious
Hoppe et al., 2001	Human	Medication-resistant epilepsy patients	VNS	–	30 Hz	500 μs	30 s/30 s	6 months	No effect on cognitive performance	ROBINS-I Serious
Klinkenberg et al., 2013	Human	Refractory epilepsy children	iVNS	0.25–0.5 mA	–	–	–	–	No significant negative effect on cognition	ROB2 Low risk
Pipan et al., 2020	Human	Drug-resistant epilepsy patients	iVNS	0.5 mA	–	250	30 s/30 s	At least 10 months	Fewer responders in cognitive deficit patients; cognitive deficit patients showed improvements of milder seizures and alertness	ROBINS-I Moderate
Puteikis et al., 2023	Human	Drug-resistant epilepsy patients	iVNS	Ramp-up output current	–	–	–	–	Improved in social cognition and short-term visual memory	ROBINS-I Serious
Shiraishi et al., 2024	Human	Epilepsy patients	taVNS	1.5 mA	25 Hz	250	30 s/30 s	20 weeks	Improved quality-of-life score	Case report
Soleman et al., 2018	Human	Epilepsy children	iVNS	NA	–	–	–	–	Improve cognitive PEDSQL Core	ROBINS-I Serious
Strong et al., 2018	Human	Refractory epilepsy	iVNS	–	5 Hz	–	–	–	Increased in delayed free recall performance	ROB2 Some concerns
Wang et al., 2016	Male SD rats	Epilepsy model	TNS	10 mA	140 Hz	–	1 min/4 min	4 weeks	Improved the cognitive impairment in epileptic rats measured by MWM	NA
Yang et al., 2023	Human	Drug-resistant epilepsy patients	taVNS	–	25 Hz	250	30 s/30 s	20 weeks	No significant cognitive difference between groups	ROB2 Low risk
**4. POCD**										
Xiong et al., 2019	Aged male SD rats	POCD	iVNS	2 V, 10 Hz, 1 ms	10 Hz	–	–	Before surgery	Improved behavioral test in MWM test, while no significant differences of OFT	NA
Zhou et al., 2022	Human	Elderly dNCR patients	taVNS	Ramp-up output current	10 Hz	300	–	1 h before anesthetic induction until the end of surgery	Incidence of dNCR decreased	ROB2 Some concerns
Zhou et al., 2023	Aged male SD rats	POCD	taVNS	1 mA	10 Hz	–	–	5 days	taVNS alleviates sevoflurane-mediated cognitive impairment	NA
**5. Cerebrovascular accidents and cranial trauma**										
Caloc’h et al., 2023	Human	TBI patient	ONS combined	–	–	–	–	–	Improved executive and cognitive functions	Case report
Choi et al., 2022	C57BL/6 mice	tBCCAO	taVNS	1 mA	20 Hz	330	–	2/6 days	Short and long-term taVNS alleviate the cognitive impairment	NA
Liu et al., 2016	SD rats	Cerebral I/R model	VNS	1 mA	20 Hz	–	3 s/3 s	10 min	Improved spatial memory in MWM test, and fear memory in automated shuttle box test	NA
Rorsman and Johansson, 2006	Human	Stroke	TENS	Ramp-up output current, 0.4 mA	2 and 80 Hz	–	–	10 weeks	Improved cognitive function	ROB2 Some concerns
Smith et al., 2005	Male Long Evans hooded rats	TBI model	iVNS	0.5 mA	20 Hz	–	–	2 weeks	Improved cognitive behaviors in MWM test	NA
Wu et al., 2018	SD rats	Cerebral I/R model	tVNS	0.8 mA	15 Hz	–	–	5 days	tVNS improved cognitive deficit after stroke indicated by MWM test	NA
Xu et al., 2023	JAX mice	TBI model	TNS	0.2 mA	40 Hz	200 μs	–	7 days	Improved cognitive functions	NA
**6. TRD**										
Desbeaumes Jodoin et al., 2018	Human	TRD	iVNS	Average stimulation intensity 1.42 mA (0.75–1.75 mA)	30 Hz	250 μs	30 s/5 min	2 years	Improved cognitive functions characterized by learning and memory	ROBINS-I Serious
Sackeim et al., 2001	Human	TRD	VNS	0.97 mA	–	–	30 s/5 min	10 weeks	Improved neurocognitive function	ROBINS-I Serious
**7. ADHD patients**										
Jonsdottir et al., 2004	Human	ADHD children	TENS	–	2 and 160 Hz	100 μs	–	12 weeks	Moderate beneficial influence on cognitive functions	ROBINS-I Serious
Loo et al., 2021	Human	ADHD children	TNS	2–4 mA	120 Hz	250	30 s/30 s	4 weeks	Increased frontal EEG power	ROB2 Low risk
**8. Cognitive impairment caused by other factors**										
Chunchai et al., 2016	SD rats	HFD model	iVNS	500 μs, 20 Hz, 0.5–0.75 mA	20 Hz	500 μs	–	12 weeks	Increased dendritic spine density and improved cognitive function	NA
McIntire et al., 2021	Human	Sleep deprivation stress	ctVNS	–	25 Hz	–	–	2 min	Improved arousal, multi-tasking, and reported performance	ROB2 Low risk
Zhang et al., 2020	Human	Chemotherapy-induced cognitive impairment	EA/TNS+BA	48 mA	2 Hz	100 μs	–	8 weeks	Improved working memory and neurological symptoms	ROB2 Low risk

AVLT-H, auditory verbal learning test-HuaShan version; aVNS, auricular vagus nerve stimulation; ctVNS, cervical transcutaneous VNS; dNCR, delayed neurocognitive recovery; EA, electroacupuncture; I/R, ischemia and perfusion; LFP, lateral fluid percussion injury; MoCA-B, Montreal cognitive assessment-basic; MWM, Morris water maze; NA, not available; NOR, novel object recognition; OFT, open filed test; ONS, occipital nerve stimulation; PAT, passive avoidance task; POCD, postoperative cognitive dysfunction; RAVNS, respiratory-gated taVNS; ROB2, revised Cochrane risk-of-bias tool for randomized trials; ROBINS-I, risk of bias in non-randomized studies–of interventions; STTB, shape trail test B; tBCCAO, transient bilateral common carotid artery occlusion; TBI, traumatic brain injury; TENS, transcutaneous electrical nerve stimulation; TNS, Trigeminal nerve stimulation; TRD, treatment-resistant depression; taVNS, transcutaneous auricular vagus nerve stimulation; tVNS, transcutaneous vagus nerve stimulation; VSEP, vagus somatosensory evoked potential.

## 2 Mechanisms of peripheral nerve stimulation

**FIGURE 2:**
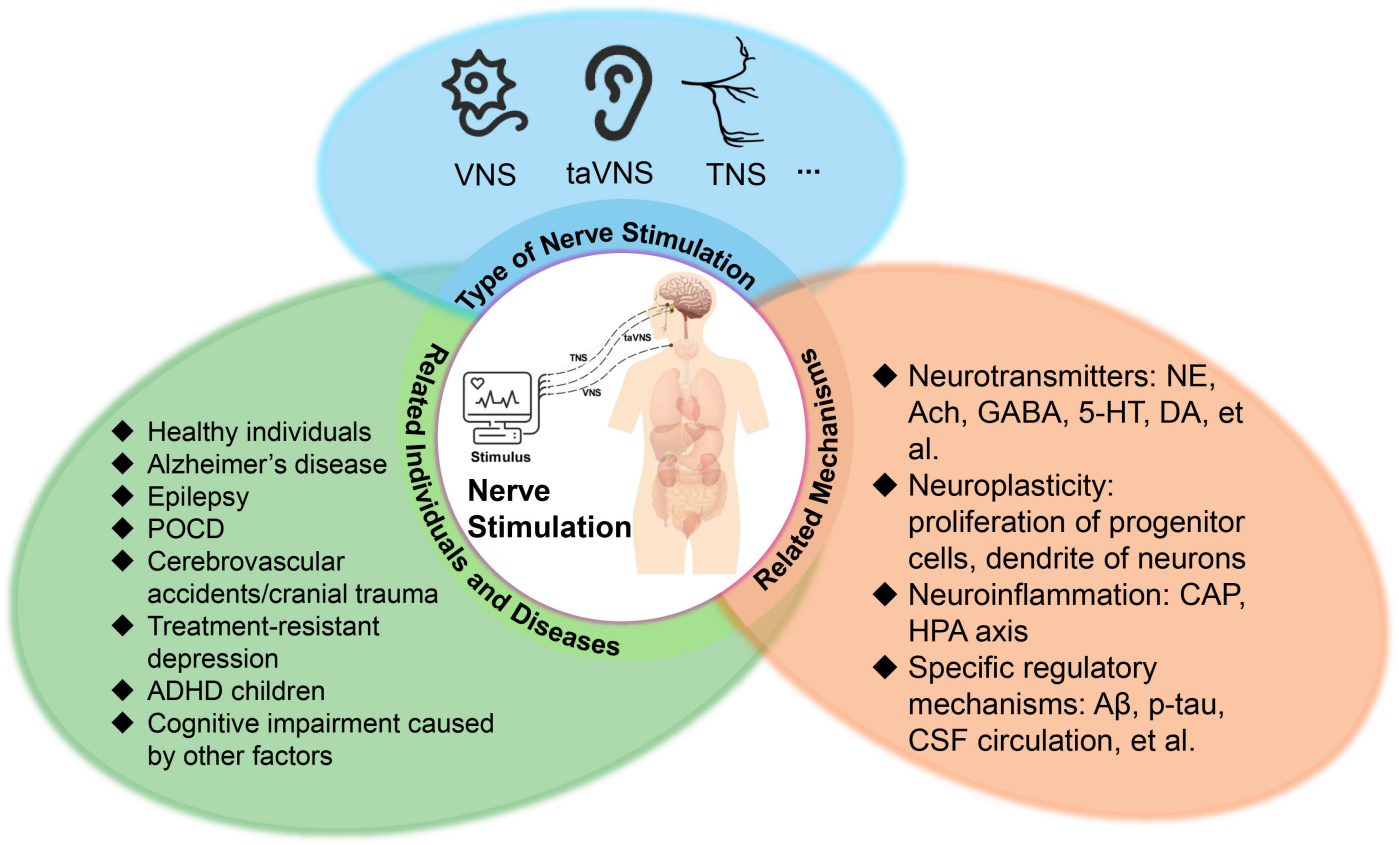
Diseases and mechanisms associated with peripheral nerve stimulation. ADHD, attention deficit hyperactivity disorder; 5-HT, 5-hydroxytryptamine; ACh, acetylcholine; CAP, cholinergic anti-inflammatory pathway; CSF, cerebrospinal fluid; DA, dopamine; GABA, γ-aminobutyric acid; HPA axis, hypothalamic-pituitary-adrenal axis; iVNS, invasive vagus nerve stimulation; NE, norepinephrine; POCD, postoperative cognitive dysfunction; taVNS, transcutaneous auricular vagus nerve stimulation; TENS, transcutaneous electrical nerve stimulation.

The mechanisms of PNS on cognitive function remain unknown. While there exists a paucity of established mechanisms for the modulation of cognitive function through PNS, our focus will be on elucidating the mechanisms of VNS on cognitive function. Broadly, VNS has been observed to influence cognitive function through various pathways, including modulation of neurotransmitter levels such as dopamine, 5-hydroxytryptamine (5-HT), acetylcholine, Gamma-aminobutyric acid (GABA), and norepinephrine. Additionally, VNS influences the functionality of cognitively relevant brain regions such as the locus coeruleus (LC), nucleus tractus solitarius (NTS), and hippocampus, while also promoting neuroplasticity and attenuating neuroinflammatory responses (Bowles et al., 2022; Slater and Wang, 2021). Mechanisms related to the effects of peripheral nerve stimulation (PNS) on cognitive function are detailed in Figure 2.

The LC serves as a key brain region associated with norepinephrine accumulation. Decreased levels of norepinephrine have been linked to heightened neuronal damage in the brains of patients with epilepsy (Larsen et al., 2023; Vespa et al., 2022). VNS has been shown to augment the putative firing activity of norepinephrine neurons by activating excitatory adrenergic receptors within the LC (Liu et al., 2016; Tseng et al., 2021). Furthermore, VNS facilitates elevated norepinephrine concentrations within critical brain areas such as the cortex, hippocampus, and medial prefrontal cortex (Hulsey et al., 2019). Additionally, VNS exerts inhibitory effects on GABAergic interneurons, which in turn alleviate the suppressive impact on norepinephrinergic neurons, thus contributing to the amelioration of depressive symptoms (Broncel et al., 2019).

In addition, neuroplasticity plays a pivotal role in AD, depression, and stroke (Duman et al., 2016; Obermeyer et al., 2019; Styr and Slutsky, 2018). Numerous studies have highlighted the beneficial effects of VNS on neuroplasticity within the brain (Bowles et al., 2022; Olsen et al., 2022). Following VNS stimulation, the dentate gyrus of the hippocampus exhibits neurogenesis or progenitor cell differentiation into fully functional neurons, accompanied by an augmentation in the dendritic length and complexity of hippocampal neurons (Gebhardt et al., 2013; Van Lysebettens et al., 2019). Moreover, VNS activation facilitates the release of norepinephrine and acetylcholine within the central nervous system (CNS), thereby promoting accelerated remodeling of the cerebral cortex post-stroke and enhancing cognitive function among patients (Gao Y. et al., 2023).

Furthermore, the vagus nerve serves as a crucial conduit for communication between the immune system and the brain. Inflammatory signaling pathways within neurons and glial cells are implicated in the progression of diseases associated with altered cognitive function, such as AD, depression, epilepsy, and stroke (Gao C. et al., 2023; Shi et al., 2019; Vezzani et al., 2019; Wu and Zhang, 2023). Among these pathways, a notable neuroimmunomodulatory mechanism, termed the cholinergic anti-inflammatory pathway (CAP), mediates communication between the central nervous and immune systems. This pathway, primarily facilitated by the vagus nerve and acetylcholine, exerts a dampening effect on the inflammatory response (Kelly et al., 2022; Qian et al., 2022). Under VNS stimulation, the α-7 nicotinic acetylcholine receptor (α7nAChR) expressed by microglia attenuates the levels of pro-inflammatory factors, such as IL-1β, IL-6, and TNF-α, thereby mitigating the inflammatory response within the brain (Liu et al., 2023; Qian et al., 2022). This attenuation is characterized by reduced levels of inflammatory cytokines and decreased apoptosis (Keever et al., 2023). Furthermore, VNS regulates the hypothalamic-pituitary-adrenal (HPA) axis, reducing levels of pro-inflammatory factors in the body and enhancing its immunoregulatory capacity. This multifaceted regulatory role of VNS is implicated in the management of conditions such as epilepsy and depression (Doerr et al., 2023; Perrin and Pariante, 2020).

Studies indicated that specific alterations were observed in certain diseases. For instance, in AD patients, one-year invasive VNS treatment led to a reduction in cerebrospinal fluid (CSF) tau protein levels, however, this was accompanied by an increase in tau protein phosphorylation (Merrill et al., 2006). In an aged rat model of postoperative cognitive dysfunction (POCD), auricular VNS (aVNS) demonstrated efficacy in mitigating the surgically induced elevation of Aβ_40_, Aβ_42_, and tau protein phosphorylation (Cai et al., 2019). Furthermore, in patients with vascular cognitive impairment (VCI), aVNS exhibited the capacity to ameliorate cognitive impairment by enhancing cerebrospinal fluid circulation (Choi et al., 2022).

In conclusion, the mechanisms underlying the beneficial effects of VNS in alleviating conditions or ameliorating cognitive impairment exhibit diversity and warrant further exploration by researchers. With ongoing advancements in relevant research techniques, it is anticipated that our knowledge and understanding of PNS will continue to evolve, thereby facilitating the wider adoption of this technique.

## 3 The role of peripheral nerve stimulation on cognition performance

### 3.1 Healthy individuals

VNS stands as an efficacious treatment across diverse neurological and psychiatric conditions. To comprehensively grasp its impact on cognitive function, delving into its effects on both cognitively impaired patients and healthy individuals is imperative, ensuring a holistic understanding.

Given the sensitivity of VNS outcomes to stimulation parameters, it is crucial to optimize responses for various clinical indications. To determine optimal parameters, four stimulation frequencies (2, 10, 25, and 100 Hz) were tested in healthy individuals using functional MRI (fMRI) on the NTS (Sclocco et al., 2020). The study found that all frequencies were effective, with 100 Hz showing the strongest brainstem responses and increased activation in monoamine neurotransmitter and pain-related nuclei.

According to conflict monitoring theory, processing situational signals like perceptual response conflict is crucial for balancing shielding and relaxation, which enhances cognitive control. To validate this theory, Fischer et al. (2018) administered left ear transcutaneous VNS to students, with average stimulation intensities of 1.3 mA in the active group and 1.49 mA in the sham group. They assessed changes in N2 and P3 event-related potentials (ERPs) and conflict task performance. The results showed reduced conflict interference and N2 and P3 amplitude post-conflict, with VNS treatment notably improving adaptation to conflict and increasing N2 amplitude (Fischer et al., 2018).

Perseverative cognitions can induce stress even without an actual stressor present. To examine the impact of non-invasive VNS on cognitive and autonomic responses to perseverative cognition after a psychosocial stress task, researchers conducted a trial using taVNS treatment on healthy individuals. The findings showed that taVNS notably decreased cognitive rigidity, as evidenced by decreased subjective perseverative thinking post-psychosocial stress (De Smet et al., 2023).

As mentioned above, research has demonstrated the beneficial impact of taVNS on cognitive function in healthy individuals, particularly in enhancing attention, memory, executive function, and cognitive control in singular tasks (Borges et al., 2020; Colzato and Beste, 2020). However, in multitasking scenarios, the concurrent processing of various stimuli creates challenges in stimulus-response (S-R) translation. Consequently, exploring the influence of taVNS on multitasking abilities becomes imperative (Logan and Delheimer, 2001). Sommer et al. (2023) recruited healthy participants aged 18–30 to engage in a dual-task paradigm under taVNS stimulation. They assessed behavioral performance, primarily focusing on reaction time, across three cognitive test blocks, and the results indicated a notable increase in inter-task interference during the initial test block, while not in the subsequent blocks during taVNS treatment (Sommer et al., 2023). While another study investigated the impact of transcutaneous VNS on the cognitive flexibility of healthy individuals, it found that tVNS may affect the LC-NE system, as indicated by the secretion of NE, dopamine, and serotonin. However, the researchers found no significant difference in task-switching ability, which referred to the ability to switch quickly between two or more cognitive tasks (Tona et al., 2020).

Previous studies have preliminarily explored the cognitive effects of various pathologies in animal models after extended VNS procedures (Wang et al., 2016; Yu et al., 2018). However, chronic VNS suffers from the shortcomings of a long treatment cycle, and the understanding of the mechanisms and effects associated with VNS can be further expanded by conducting studies related to acute VNS in animal models. Research revealed that acute VNS treatment enhanced short-term memory and cognitive flexibility in naïve rats, with no significant correlation between cognitive effects and VNS-induced non-specific alterations in motor functions or anxiety levels (Driskill et al., 2022). Furthermore, following a single 30-min session of invasive VNS, stimulated rats showed enhanced performance in behavioral tests, along with increased long-term potentiation (LTP), spontaneous spike amplitude, frequency, and elevated brain-derived neurotrophic factor (BDNF) levels in the CA1of hippocampus (Olsen et al., 2022). The above studies demonstrated that chronic VNS could enhance cognition and task processing, while short-term VNS may also improve cognitive flexibility, supporting the promotion of this promising treatment modality.

Cognitive studies have implicated epigenetic mechanisms such as RNA-DNA-protein interactions, DNA methylation, and chromatin remodeling to effective learning and memory (Breen et al., 2024; Wilson et al., 2023). Thus, Sanders et al. (2019) examined the epigenetic changes in hippocampal and cortical regions following VNS treatment (100 μs biphasic pulses, 30 Hz, 0.8 mA) in healthy rats for 4 days to elucidate the mechanisms underlying VNS effects on cognitive functions. They observed a significant correlation between performance in the novelty preference test (NPTP) and epigenetic changes, specifically reduced HDAC11 levels in the hippocampus and elevated IEG ARC levels in the cerebral cortex. These changes, linked to plasticity genes such as HDAC3 and transcriptional regulators, implicate histone acetylation and alterations in hippocampal and cortical HDAC in VNS-mediated modulation of stress response, neuronal plasticity, and memory consolidation.

To improve working memory while mitigating taVNS-related deficiencies, recent researchers developed a novel and practical vibrotactile taVNS system. The results suggest that 6 Hz vibrotactile auricular VNS holds promise as an intervention for enhancing working memory performance as it may boost general arousal and counteract decreases in arousal during continuous working memory tasks in healthy individuals (Tan et al., 2024).

### 3.2 Alzheimer’s disease and related cognitive impairment

AD is a multifaceted neurodegenerative disorder impacting over 30 million people globally. It is influenced by factors such as age, gender, genetics, lifestyle, gut microbiota, and personal brain disease history (Hu et al., 2021; Mazure and Swendsen, 2016; Rajendran and Krishnan, 2024). Traditional single-mechanism interventions have shown limited efficacy, prompting the exploration of novel strategies, including nano-drug delivery systems, immune response modulation for amyloid clearance, and neuron replacement with stem cells (Almuhayawi et al., 2020; Cone et al., 2021; Song et al., 2022). While these emerging approaches show promise, they require further clinical validation. AD is characterized by multiple pathological mechanisms, including Tau hyper-phosphorylation (Chiasseu et al., 2017; Goedert et al., 1988; Hausrat et al., 2022), mitochondrial dysfunction (Wang et al., 2020; Zhang et al., 2023), synaptic loss (Forner et al., 2017; Pereira et al., 2021; Zhong et al., 2023), and cholinergic system impairment (Giacobini et al., 2022; Hampel et al., 2018; Schumacher et al., 2022). Research indicates that somatosensory stimulation may enhance hippocampal activity and acetylcholine release61, whereas VNS could improve motor refinement via cholinergic signaling (Bowles et al., 2022).

Previous research on VNS in AD patients has produced conflicting results. One open-label pilot study indicated that VNS can mitigate cognitive decline after 3 months of treatment, with 7 out of 10 patients showing improvement assessed by Alzheimer’s Disease Assessment Scale-cognitive subscale (ADAS-cog, median improvement of 3.0 points) and 9 out of 10 patients showing improvement assessed by Mini-Mental State Examination (MMSE, median improvement 1.5 points). After 6 months of treatment, a similar effect of improved cognitive function was demonstrated (Sjögren et al., 2002). Merrill et al.’s (2006) study showed no significant cognitive decline in 12 out of 17 patients after 1 year of VNS treatment measured by MMSE and ADAS-cog. However, other studies have reported limited cognitive improvement.

Mild cognitive impairment (MCI) frequently precedes dementia, manifesting as relatively preserved daily functioning alongside documented cognitive decline. The transition from MCI to dementia is conservatively estimated at 5%–10% annually, with comparable rates observed for regression from MCI to normal cognition. Thus, MCI is an unstable cognitive state, and effective interventions for this group of patients have the potential to reduce the transition to dementia and mitigate the ensuing social sequelae. Luijpen et al. (2004) conducted two independent studies focusing on the effects of transcutaneous electrical nerve stimulation (TENS) on older patients with MCI, one study delved into self-efficacy and mood, and the other centered on memory (location: between the 1st and 5th thoracic level on each side of the spinal column) (Luijpen et al., 2005). TENS treatment showed moderate improvement in cognitive function (measured by the MMSE scale), self-efficacy (measured by the Groninger Activity Restriction Scale and Philadelphia Geriatric Center Morale Scale), and mood (measured by the Geriatric Depression Scale). It is notable that while previous research has indicated that TENS (location: on the back between Th1 and Th5) has a positive impact on memory function in AD patients, particularly those in the early stages (Scherder and Bouma, 1999), Luijpen et al.’ (2005) study revealed that for individuals with MCI, TENS did not enhance their memory capacity. The reason for this paradoxical result may be the distinct responses of the cholinergic system to TENS at different stages of the disease course of AD-related cognitive impairment. Primarily, ChAT activity levels in the CSF, prefrontal cortex, and hippocampus of MCI patients surpass those of both normal elderly individuals and AD patients (DeKosky et al., 2002; Karami et al., 2021). TENS, through its stimulation of cholinergic basal forebrain neurons, might intensify activation in the prefrontal cortex and hippocampus. Essentially, this could lead to an overactivation of the cholinergic system, potentially yielding ineffective or even adverse outcomes.

Given that individuals with MCI possess a dual potential for transitioning either toward dementia or retaining normal cognitive function, interventions at this stage hold significant clinical importance. Consequently, recent research has predominantly concentrated on patients with MCI, particularly exploring VNS. Wang et al. (2022) demonstrated noteworthy alterations in cognitive function rating scales, such as Montreal cognitive assessment-basic (MoCA-B), auditory verbal learning test-HuaShan version (AVLT-H), and shape trail test (STTB), following 24 weeks of taVNS treatment for MCI patients. Furthermore, employing fMRI techniques, researchers observed alterations in functional connectivity among brain regions crucial for semantic and salience functions, notably involving temporal and parietal regions, in MCI patients undergoing tVNS. Additionally, connectivity originating from the hippocampus to various cortical and subcortical regions of interest (ROI) clusters exhibited changes with tVNS compared to ear lobe stimulation. In summary, tVNS induced modifications in brain region connectivity networks associated with the progression of AD, thereby providing insights for potential treatment strategies or mitigation of AD-related cognitive impairments (Murphy et al., 2023).

Although the results of available studies have been inconclusive, a synthesis of relevant studies on VNS and AD indicates that VNS may be an effective method of preventing further deterioration of cognitive function or even enhancing it. However, the impact of different AD disease stages on VNS efficacy should also be fully considered when applying it to clinical practice.

In addition to VNS, TENS has also been reported as a therapeutic option for addressing cognitive dysfunction in AD patients. To investigate the impact of TENS on cognitive function, Scherder et al. (1992, 1998, 2000) conducted a series of studies. Initially, they examined the effects of TENS treatment on patients with Alzheimer’s-type dementia, revealing a significant improvement in verbal long-term memory. However, no significant effects were observed on visual long-term memory, as well as verbal and non-verbal short-term memory (Scherder et al., 1992). Subsequently, considering potential environmental influences on treatment outcomes, they explored the efficacy of TENS in AD treatment when administered without therapists. This investigation also demonstrated cognitive enhancement with this treatment protocol (Scherder et al., 1998). Finally, they investigated the cognitive and behavioral effects of TENS in non-demented older adults, revealing improvements in visual short-term and verbal long-term (recognition) memory, as well as semantic verbal fluency. Additionally, these older adults were less likely to exhibit depressive-like moods (Scherder et al., 2000). However, another randomized controlled trial showed that in aged AD patients, TENS treatment (100 μs biphasic pulses, 160 Hz, location on the back at the first thoracic vertebra) did not result in significant changes in cognitive functioning as measured by Digit Span, Face Recognition on the Rivermead Behavioral Memory Test, and the Eight Word Test on the Amsterdam Dementia Screening Test (van Dijk et al., 2005).

In particular, it is observed that the majority of the studies included in this review, which investigate the effects of PNS in this demographic, primarily concentrate on individuals with MCI or AD. However, these studies do not extensively examine the underlying mechanisms through which PNS might influence cognitive function in these conditions. Additionally, the studies are generally characterized by short durations, with the maximum follow-up period extending to only one year. These limitations impede a comprehensive understanding of the therapeutic potential of PNS in AD and constrain its broader clinical application. Nonetheless, the extant evidence indicates that PNS may represent a promising approach to mitigating cognitive decline associated with AD and related disorders.

### 3.3 Epilepsy

Epilepsy is a common chronic neurological disease of the CNS, which is featured by recurrent unprovoked seizures. According to the latest research on the global burden of neurological diseases, epilepsy ranks among the top ten conditions with the highest age-standardized DALYs in 2021 (GBD 2021 Nervous System Disorders Collaborators, 2024). Despite the approval and widespread adoption of innovative antiseizure medications (ASMs) over recent decades, up to one-third of individuals with epilepsy (PWE) continue to experience seizures despite treatment (Sultana et al., 2021). As epilepsy progresses, some patients may experience cognitive impairment. Given its efficacy in treating CNS disorders, VNS has been successfully used in patients with refractory epilepsy, including pediatric patients (Drees et al., 2024; Haddad et al., 2023; Seth et al., 2024). As VNS influences the functioning of specific brain regions, it’s crucial also to consider its potential effects on cognitive functioning when administering VNS therapy to patients with epilepsy.

In a clinical study investigating the impact of VNS on cognition and quality of life, it was found that after 12–14 weeks of treatment, VNS administered at higher stimulus intensities (as used clinically) did not yield significant alterations in cognitive functioning in either group. However, patients receiving higher stimulus intensities (as used clinically) reported experiencing fewer emotional and physical issues compared to those receiving lower stimulus intensities (minimal intensity) (Dodrill and Morris, 2001). Yet, a recent case report of two young female epilepsy patients showed that after 20 weeks of taVNS therapy, both achieved seizure freedom and improved quality-of-life scores (Shiraishi et al., 2024). Interestingly, the role of the VNS showed inconsistent effects on cognition when multiple cognitive function rating scales were included. Ghacibeh et al. (2006) compared the effects of actual and sham stimuli delivered by an implantable VNS device across various cognitive tasks, including assessments of cognitive flexibility, creativity, and memory. The results indicated that while VNS impaired cognitive flexibility and creativity, it did not negatively impact learning, and even enhanced attention.

Epilepsy is reported to be the third leading cause of DALYs among children and adolescents aged 5–19 years (GBD 2021 Nervous System Disorders Collaborators, 2024). A study of VNS in children with refractory epilepsy showed that after VNS treatment, seizure frequency resolved in 6/15 of the children, seizure completely resolved in one of the children, and QOL scores improved in 12 others; however, VNS did not appear to have a significant effect on cognitive function (Hallböök et al., 2005). Similarly, a randomized controlled study on children with intractable epilepsy found that after 20 weeks of iVNS treatment, while cognitive function remained unchanged, seizure frequency decreased, mood improved, and depression scores significantly lowered (Klinkenberg et al., 2013). Soleman et al. (2018) conducted a comparative analysis of children with epilepsy (aged < 12) undergoing VNS implantation and found no overall cognitive function changes; however, subgroup analysis showed significant cognitive improvement when VNS was implanted before age 5.

The researchers further investigated the impact of VNS on cognitive function in adults with medication-resistant epilepsy. Neuropsychological assessments were conducted both pre- and at least 6 months post-implantation of the stimulation device. Interestingly, the cognitive function evaluations revealed no significant differences in attention, learning and memory capacity, or short-term memory, and no adverse effects were observed after VNS (500 μs biphasic pulses, 30 Hz) (Hoppe et al., 2001). Furthermore, another study examining the effects of taVNS (250 μs biphasic pulses, 25 Hz) over 20 weeks in patients with drug-resistant epilepsy revealed no significant alterations in Montreal Cognitive Assessment (MoCA) scores, QOL scores, or mood scores [including measures such as Hamilton Anxiety Scale (HAMA), Hamilton Depression Scale (HAMD), and Mini-International Neuropsychiatric Interview (MINI)]. Additionally, participants reported discomfort in the form of pain, sleep disturbances, and flu-like symptoms (Yang et al., 2023).

Two research groups investigated the effects of VNS on patients with drug-resistant epilepsy, comparing long-term [ Puteikis et al. (2023), lasting at least 24 months] and short-term [ Strong et al. (2018), 12 months] studies. After excluding participants with emotional and cognitive dysfunction, patients underwent treatment with different VNS frequencies. Theta frequency stimulation resulted in a 23% improvement in free recall, while beta and theta frequencies led to 5% and 15% reductions in accuracy and reaction time, respectively. In the short-term study, only one patient was ultimately enrolled. Meanwhile, a separate study on prolonged iVNS showed improvements in social cognition and short-term visual memory, as demonstrated by enhanced performance on the ROCF and Happé Strange Stories Test.

Patients with concurrent cognitive dysfunction and epilepsy often have additional neuropsychiatric conditions, such as cerebral palsy and autism spectrum disorder. Drug resistance in this group is notably high, around 45%. Although a retrospective study on patients with cognitive dysfunction and epilepsy undergoing iVNS therapy found that response rates were lower in those with both conditions, significant improvements in seizure reduction and alertness were noted (Pipan et al., 2020).

In addition to investigating VNS for epileptic patients, Wang et al. (2016) constructed a rodent epilepsy model by administering pilocarpine to explore the impact of another type of PNS-Transcutaneous nerve stimulation (TNS)-on epilepsy pathogenesis and cognitive function. The study’s findings revealed that immediate TNS treatment following the onset of epileptic seizures not only mitigated chronic spontaneous seizures but also ameliorated cognitive dysfunction in rats. These observed improvements may be attributed to TNS’s inhibitory effects on hippocampal apoptosis and the pro-inflammatory response (Wang et al., 2016).

The majority of studies included in the analysis of epilepsy treatments employed iVNS, which has been demonstrated to effectively reduce seizure frequency and, in certain instances, enhance cognitive function (Dodrill and Morris, 2001; Ghacibeh et al., 2006). Conversely, research examining non-invasive VNS in epilepsy patients has not consistently yielded significant cognitive improvements (Yang et al., 2023). The underlying reasons for this discrepancy between stimulation modalities remain unclear; however, they may be attributed to variations in stimulation parameters, the depth of nerve activation, or patient characteristics. This observation underscores the necessity for further investigation into the mechanisms driving these differences and advocates for the optimization and advancement of non-invasive VNS strategies, especially for epilepsy patients with concurrent cognitive impairments.

### 3.4 Postoperative cognitive dysfunction

Postoperative cognitive dysfunction (POCD) is a neurological complication that can occur after surgery, resulting in cognitive decline due to neuroinflammation and oxidative stress triggered by the surgical procedure. Those affected often experience a debilitating condition. Individuals with POCD face an elevated risk of developing AD compared to those who age normally.

In a prospective double-blind randomized controlled trial conducted by Zhou et al. (2022), it was discovered that taVNS administered one hour before surgery and continued until the end of surgery effectively reduced the occurrence of delayed neurocognitive recovery (dNCR) in elderly patients one week after total joint arthroplasty. The incidence rate was notably lower in the taVNS group (10%) compared to the surgical group (27.1%). The study revealed significant reductions in neuroinflammation-related factors, such as IL-6, as well as markers of cholinesterase activity including AChE and BChE, in the serum of patients receiving taVNS. Furthermore, rodent experiments were carried out to investigate the mechanism through which taVNS mitigates postoperative cognitive impairment induced by the anesthetic drug sevoflurane in aged rats. The researchers concluded that taVNS attenuated sevoflurane-induced hippocampal neuronal apoptosis, necrosis, and microglia activation by activating the basal cholinergic system in the forebrain (Zhou et al., 2023). Another research team conducted a comparable study investigating the impact of preoperative VNS for 30 min on cognitive function in elderly rats using a POCD animal model induced by splenectomy (Xiong et al., 2019). Behavioral findings indicated a noteworthy extension in the avoidance latency of the Morris water maze (MWM) among rats treated with VNS, while no significant variance was observed in the results of the open field test (OFT). Subsequent investigation uncovered that surgery and anesthesia led to elevated levels of TNF-α and IL-6 in the serum, whereas VNS intervention mitigated the levels of these inflammatory factors. In addition, recent studies have revealed that auricular VNS exhibits the potential to ameliorate POCD-related neurological impairments in aged rats subjected to laparotomy. Notably, MWM test assessments indicated reduced swimming latency and distance, alongside significant improvements in neuroinflammation-related biomarkers, such as TNF-α, NF-κB, and IL-1β. Particularly noteworthy are the observed reductions in factors associated with the progression of AD, including attenuated tau phosphorylation at AT-8 and Ser396, and decreased levels of Aβ_40_ and Aβ_42_.

In summary, both clinical observations and mechanistic investigations underscore the promising role of VNS in mitigating POCD, offering optimistic prospects for perioperative cognitive preservation amid the prevalent occurrence and associated risks of POCD.

### 3.5 Cerebrovascular accidents and cranial trauma

Traumatic Brain Injury (TBI) is a result of external force impacting the brain, standing as a primary cause of neurological disability and mortality globally. TBI encompasses various severity levels, including concussion and mild traumatic brain injury (mTBI), each capable of inducing cognitive deficits and impairment. Research indicates that approximately 65% of moderate to severe TBI patients experience enduring cognitive challenges, with up to 15% of mild TBI patients grappling with persistent cognitive impairment (Rabinowitz and Levin, 2014). However, the intricate nature of TBI poses challenges to effective treatment modalities, limiting the efficacy of current approaches in addressing and rehabilitating cognitive dysfunction (Galgano et al., 2017). Therefore, pursuing therapeutic interventions capable of ameliorating cognitive function in TBI patients holds paramount clinical significance.

Caloc’h et al. (2023) reported a case report involving a middle-aged male experiencing persistent cognitive dysfunction and intractable headaches following Traumatic Brain Injury (TBI). The utilization of bilateral Occipital Nerve Stimulation (ONS) in conjunction with multisite transcranial magnetic stimulation and cognitive training (cogT) yielded notable improvements in cognitive function, refractory pain, and depression in patients with TBI (Caloc’h et al., 2023). To elucidate the mechanism underlying the amelioration of cognitive function in TBI through neurostimulation, researchers conducted preliminary investigations using a rodent TBI model.

In a lateral fluid percussion injury (LFP) model of TBI, 14 days of VNS improved cognitive function in rats during the MWM test, but neurophysiological examination showed no significant changes in hippocampal lesions or neuron density (Smith et al., 2005). In a separate study, mice with TBI treated with TNS for 7 days showed improved cognitive function, with TNS enhancing hippocampal connectivity via corticotropin-releasing hormone (CRH) neurons in the paraventricular nucleus (PVN) and dopamine transporter (DAT) neurons in the substantia nigra compacta/ventral tegmental area (SNc/VTA) (Xu et al., 2023). These findings shed light on the specific mechanism by which TNS alleviates cognitive dysfunction in TBI.

To investigate the mechanism of taVNS in alleviating cognitive impairment from cerebrovascular disease, researchers used taVNS on an animal model of vascular cognitive impairment induced by transient bilateral common carotid artery occlusion (tBCCAO). Both short-term (2 days) and long-term (6 days) taVNS improved cognitive function and cerebrospinal fluid circulation, suggesting that taVNS enhances CSF flow and may effectively treat ischemic cognitive impairment (Choi et al., 2022). Given the close association between cholinergic neuroproteins and cognitive function, Wu et al. (2018) investigated whether tVNS could improve post-stroke cognitive dysfunction by modulating the non-neuronal cholinergic system in a rat middle cerebral artery occlusion (MCAO) model. They found that five days of tVNS alleviated cognitive impairment and altered key cholinergic factors in the hippocampus. Similarly, Liu et al. (2016) reported that VNS enhanced spatial and fear memory in a rat MCAO/R model, suggesting its potential to alleviate cognitive dysfunction, possibly through norepinephrine modulation.

Clinical studies on PNS’s impact on cognitive function post-stroke are limited, but one study showed significant cognitive improvements in patients with moderate-to-severe stroke-induced impairment after a 10-week, 20-session TENS regimen. Improvements were noted in verbal and visual memory, attention, and perception, while mood remained unaffected (Rorsman and Johansson, 2006). These findings suggest that PNS may benefit cognitive rehabilitation in ischemic stroke and cerebrovascular accident patients, though its mechanism remains unclear.

### 3.6 Treatment-resistant depression

Major Depressive Disorder (MDD) is a widespread and severe mental illness linked with considerable morbidity and mortality. The World Health Organization predicts that by 2030, MDD will be a leading cause of global disease burden (Collins et al., 2011). Individuals with MDD often experience impairments in learning, memory, and executive functioning, and many do not achieve functional remission despite various treatments (Rosenblat et al., 2016). Thus, proactive intervention strategies are essential, particularly for those who do not respond to pharmacotherapy.

Following 10 weeks of VNS treatment for Treatment-Resistant Depression (TRD), a study by Sackeim et al. (2001) found no decline in neurocognitive function, with improvements in language, executive function, and psychomotor function compared to baseline. Similarly, a long-term study of VNS in TRD patients observed significant enhancements in learning and memory after one month, with cognitive improvements persisting for two years. Initially, there was no correlation between cognitive and depression scores, but a significant correlation developed after one year of treatment (Desbeaumes Jodoin et al., 2018).

### 3.7 ADHD children

The prevalence of Attention Deficit/Hyperactivity Disorder (ADHD) among children aged 4–17 years ranges from 5% to 11%, exerting not only a negative impact on the patient’s quality of life but also imposing a significant financial burden on their families (Kessler et al., 2006; Solanto et al., 2009). Despite pharmacotherapy being the most commonly employed treatment strategy for ADHD, it is fraught with challenges such as poor adherence and adverse effects. Previous studies have indicated that TNS may offer efficacy in alleviating ADHD symptoms. Loo et al. (2021) specifically investigated the response to TNS in children with ADHD exhibiting executive dysfunction. Their findings demonstrated that this subgroup of children exhibited a favorable response to TNS, characterized by altered brain activity in the right frontal lobe, normalization of executive functioning, and a reduction in ADHD symptoms. Moreover, a prior study suggested that TENS (location: on the patient’s back between Th1 and Th5) may modestly improve cognitive functioning in children with ADHD, particularly in terms of executive functioning (Jonsdottir et al., 2004).

### 3.8 Cognitive impairment caused by other factors

To address the public health issue of fatigue, non-invasive neuromodulation, specifically cranial transcutaneous VNS (ctVNS), was tested by McIntire et al. (2021) on 40 participants who underwent 34 h of wakefulness. The ctVNS group showed improved arousal, multitasking, and reported lower fatigue levels compared to the sham group, indicating ctVNS may effectively reduce fatigue (McIntire et al., 2021).

Epidemiological studies reveal that over 75% of cancer patients experience acute cognitive decline during chemotherapy, with approximately 17%–34% developing long-term cognitive impairment (Hede, 2008). Findings from a randomized controlled trial conducted by Zhang et al. (2020) demonstrated that electroacupuncture trigeminal nerve stimulation combined with body acupuncture (EA/TNS+BA) significantly enhanced cognitive performance levels in breast cancer patients undergoing chemotherapy. Specifically, after 2 and 8 weeks of treatment, patients exhibited significantly improved performance on the Reverse Digit Span Test. Moreover, the incidence of adverse events such as diarrhea, loss of appetite, headache, anxiety, and irritability significantly decreased. However, no significant changes were observed in MoCA scores or the incidence of chemobrain.

Animal model studies investigating cognitive dysfunction have demonstrated that VNS mitigates cognitive decline in obese insulin-resistant rats. This effect is attributed to VNS’s ability to alleviate mitochondrial dysfunction, enhance insulin sensitivity in the brain, increase dendritic spine density, and reduce cell apoptosis (Chunchai et al., 2016).

## 4 Conclusion

The existing studies have shed light on certain aspects of how PNS affects cognitive function (Figure 2). Collectively, recent findings regarding PNS indicate potential cognitive benefits for healthy individuals, encompassing enhancements in learning, memory, and executive function. However, the impact of VNS and TENS on cognitive function across various neuropsychiatric conditions is notable variability. In AD, MCI, cerebrovascular disease, and other scenarios involving factors like surgery/anesthesia, dietary influences, or additional comorbidities affecting cognition (e.g., depression), VNS may enhance cognitive abilities, although findings across studies have been somewhat inconsistent. Conversely, for conditions like epilepsy and ADHD where cognitive impairment is less pronounced, VNS demonstrates notable efficacy in symptom alleviation and quality of life enhancement but does not substantially affect cognitive function. Consequently, based on existing research, we posit that PNS may enhance cognitive function to a certain extent in subjects, or at the very least, it does not appear to exacerbate cognitive decline.

## 5 Expert opinion

In clinical practice, the options for enhancing cognitive function are limited, with PNS being one of the few available interventions. However, the efficacy of PNS on cognitive performance is influenced by multiple factors, including the specific parameters of the stimulation and the underlying characteristics of the patient population, such as the type of disease. Based on a comprehensive review of the current literature, we propose that future research should prioritize addressing the following key considerations. Firstly, attention must be directed toward understanding potential gender disparities in response to PNS. As our understanding of diseases deepens and genetic engineering-related technologies evolve, it has become evident that conditions like AD and depression are influenced by gender (Barth et al., 2023; Seney et al., 2022; Yan et al., 2022). Yet, current research on the effects of various PNS techniques on both diseases and cognitive performances inadequately addresses gender differences, warranting future attention in this regard. Secondly, the vagus nerve plays a crucial role in the CAP, acting as a pivotal conduit for peripheral nerve inflammation and CNS inflammation. The intestinal flora, serving as a key initiator of peripheral neuroinflammation, not only produces various neurotransmitters such as dopamine and serotonin but also triggers peripheral neuroinflammation by releasing lipopolysaccharides into the bloodstream through intestinal permeability. Consequently, the intestinal flora is believed to exert a significant influence on disorders characterized by cognitive impairment (Dalile et al., 2019; Sarkar et al., 2018). Furthermore, given the close association between PNS, neurotransmitters, and neuroinflammation, it is conceivable that PNS may also impact cognitive function through modulation of the intestinal flora. However, no studies have yet explored this potential avenue. Finally, owing to the unclear mechanism of action and the occurrence of certain side effects associated with PNS, some patients require long-term or repeated hospitalizations, placing a considerable burden on families and society alike. This presents a significant obstacle to the widespread adoption of this therapeutic approach. Therefore, the development of user-friendly, portable devices may serve to facilitate the broader implementation of this promising therapeutic strategy.
