# Supplementary figures and images for: Associations between peripheral nerve stimulation and cognitive performance: insights from healthy individuals and various disease pathologies
Source: Front Aging Neurosci. 2025 Sep 2;17:1518198. doi: 10.3389/fnagi.2025.1518198 (PMC12436292; doi:10.3389/fnagi.2025.1518198)

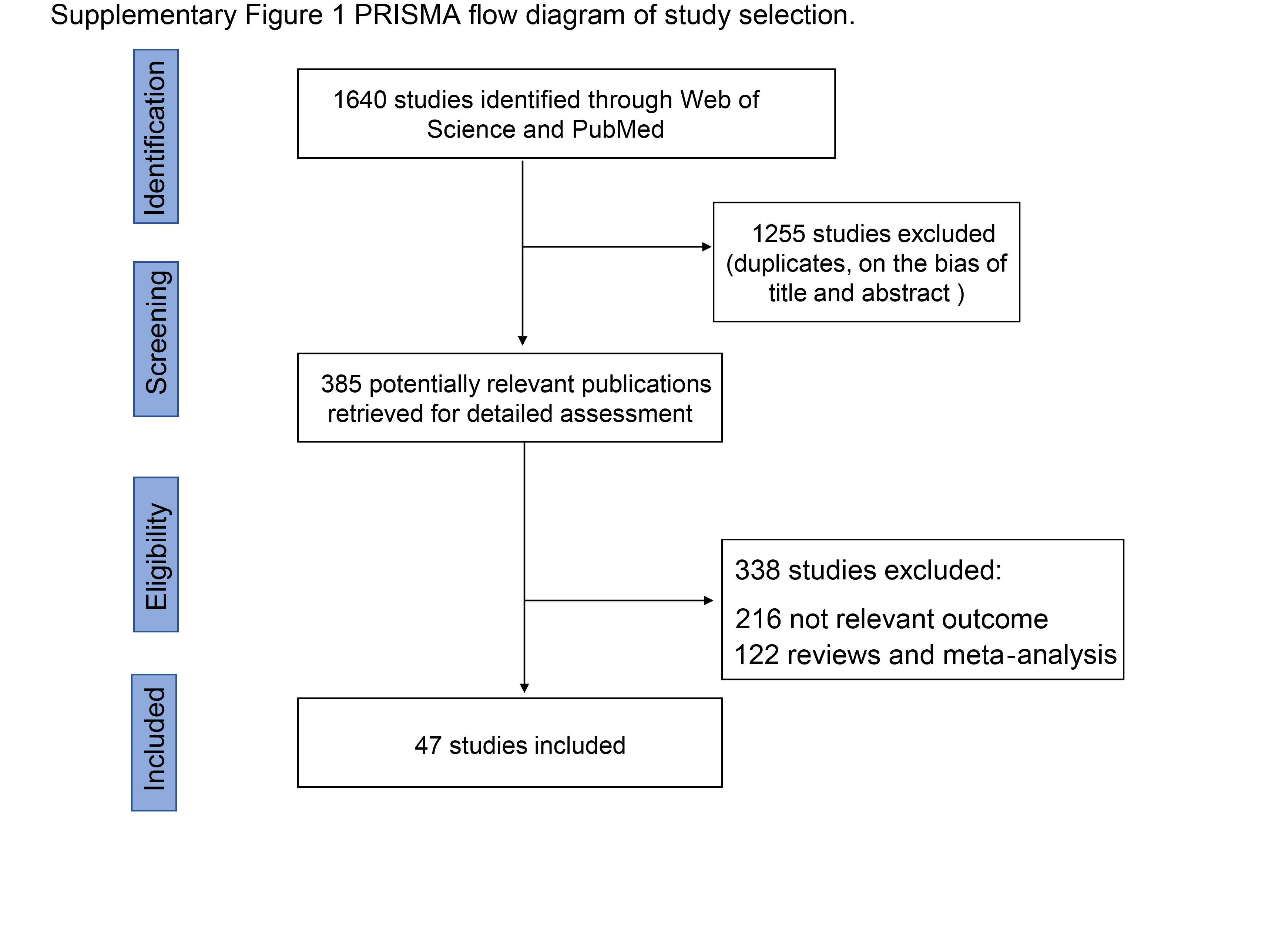

Supplement: Supplementary file 1 [file Image_1.jpeg]
